# Genomes and geography: genomic insights into the evolution and phylogeography of the genus *Schistosoma*

**DOI:** 10.1186/1756-3305-4-131

**Published:** 2011-07-07

**Authors:** Scott P Lawton, Hirohisa Hirai, Joe E Ironside, David A Johnston, David Rollinson

**Affiliations:** 1 The Institute of Biological, Environmental & Rural Sciences, Aberystwyth University, Penglais, Aberystwyth, Ceredigion, SY23 3D, UK; 2 Wolfson Wellcome Biomedical Laboratories, Dept. of Zoology, Natural History Museum, London, SW7 5BD, UK; 3 The Primate Research Institute, University of Kyoto, Inuyama, Aichi 484-8506, Japan; 4 School of Medicine, University of Southampton, Mailpoint 12, Level B, Lab and Path Block, Southampton General Hospital, Southampton SO16 6YD, UK; 5 School of Life Sciences, Kingston University London, Penrhyn Road, Kingston upon Thames, Surrey KT1 2EE, UK

## Abstract

Blood flukes within the genus *Schistosoma *still remain a major cause of disease in the tropics and subtropics and the study of their evolution has been an area of major debate and research. With the advent of modern molecular and genomic approaches deeper insights have been attained not only into the divergence and speciation of these worms, but also into the historic movement of these parasites from Asia into Africa, via migration and dispersal of definitive and snail intermediate hosts. This movement was subsequently followed by a radiation of *Schistosoma *species giving rise to the *S. mansoni *and *S. haematobium *groups, as well as the *S. indicum *group that reinvaded Asia. Each of these major evolutionary events has been marked by distinct changes in genomic structure evident in differences in mitochondrial gene order and nuclear chromosomal architecture between the species associated with Asia and Africa. Data from DNA sequencing, comparative molecular genomics and karyotyping are indicative of major constitutional genomic events which would have become fixed in the ancestral populations of these worms. Here we examine how modern genomic techniques may give a more in depth understanding of the evolution of schistosomes and highlight the complexity of speciation and divergence in this group.

## Introduction

Members of the genus *Schistosoma *are digenean (Strigeiformes: Schistosomatidae) blood flukes of mammals, comprising of 23 known species, with at least 7 of these contributing to the neglected medically important disease schistosomiasis [1]. It is estimated that more than 200 million people are infected in 74 countries throughout the tropics and subtropics [1]. The evolutionary history of these parasites has generated much interest, especially with regard to understanding the relative roles that particular species have in causing human disease [1]. With the advent of molecular data, ranging from chromosomal morphology and small gene sequences to the annotation of full genomes, several theories have been put forward as an alternative to the "out of Africa" hypothesis [1-3]. This review aims to discuss some of the evidence generated from molecular biology and genomics that has contributed to the now generally accepted "out of Asia" hypothesis, but also aims to provide alternative theories to some of the established concepts in schistosome genome evolution.

### Biogeography and evolutionary origins of the genus *Schistosoma*

*Schistosoma *species are found throughout the tropical and developing regions of the world, with primary foci in Asia, Africa and South America. The distribution of these parasites is closely linked with the geography of the obligate intermediate snail hosts that each species has adapted to exploit within given regions [4,5]. The species of *Schistosoma *were originally categorised into four major groups based on distribution, host specificity and egg morphology [4,6]. There are two groups primarily found throughout Asia; the *S. japonicum *group found throughout eastern and south eastern Asia, including China, the Philippines and Malaysia, and the *S. indicum *group which inhabits the western and southern regions including India, Sri Lanka and Thailand [4,7,8]. Both *S. mansoni *and *S. haematobium *groups are found throughout Africa, with species from both groups often sharing overlapping geographical ranges [6,8]. However, the only representative of the genus *Schistosoma *to be found in South America is *S. mansoni*, which is mainly restricted to areas of Brazil, Venezuela, Surinam and the Caribbean.

**Figure 1:**
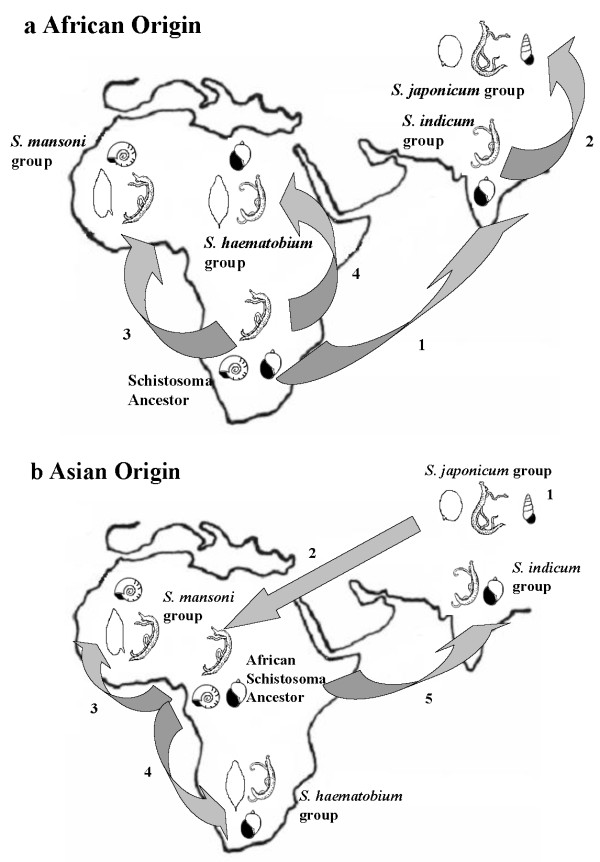
**Maps displaying the two hypotheses of the origins of the schistosomes**. 1a showing the African origin as suggested by Davis [2,3] 1) and 2) indicate the African *Schistosoma *moving over to Asia on the Indian plate 70-148 MYA, giving rise to the *S. indicum *group and diversifying into the *S. japonicum group*. 3) and 4) suggest the *Schistosoma *ancestor remaining in Africa diverged > 120 MYA, giving rise to the *S. mansoni and S. haematobium *groups. 1b showing the Asian origin as put forward by Rollinson *et al*. [6], Snyder and Loker [13]: 1) *S. japonicum*-like ancestor arises and diversifies in Asia. 2) Asian descendents of the ancestral schistosome move into Africa with the widespread mammal migration between 12-19 MYA. 3) and 4) the African schistosome diverges 1-4 MYA, giving rise to the *S. mansoni *and *S. haematobium *groups. At this time, the ancestors of the *S. indicum *group emerge and move to India again via the mass movement of large mammals (Figure adapted from [5]).

Many theories on the origin of these parasites have been put forward, developed and reviewed, with primary arguments for both an African and Asian origin [2-8]. Davis [2,3] proposed that the genus *Schistosoma *arose before the separation of the super continent Gondwanaland (which was made up of what is today Africa, South America, Antarctica and Australia) more than 150 million years ago (MYA) and had already begun to exploit pulmonate and pomatiopsid snails, of which extensive fossil records suggest a Gondwanan origin. This implies that the spread of these parasites was due to continental drift and that the ancestor of the Asian schistosomes was carried across to Asia as India separated from Africa and moved towards Asia 70-148 MYA, giving rise to the *S. indicum *and *S. japonicum *groups [2]. This theory also suggests that the remaining African stock began to radiate over 120 MYA, giving rise to the ancestral lineages of the *S. mansoni *and the *S. haematobium *groups. This would have meant that the *S. mansoni *appeared approximately 80 MYA in order for both the parasite and it's snail vector *Biomphalaria *to invade and colonise South America before the fragmentation of Gondwanaland [3,5] (Figure 1).

More recently, arguments were put forward for an Asian origin for the schistosomes [5,6,9-12]. This theory suggests that the ancestral stock of Asian schistosomes may have originally had a pomastiopsid or a pulmonate snail host. Snyder & Loker [13] related the radiation of the *S. japonicum *group to that of their pomatiopsid hosts in the mid-Miocene. If this was the case, it would indicate that the schistosomes colonised Africa approximately 15-20 MYA, almost 70 million years after the separation of the South American and African plates, as Gondwanaland drifted apart. Upon invasion of the African continent, the parasites evolved to exploit pulmonate snails exclusively, thus developing a more specialised host range [5,10,13] (Figure 1)

### *Schistosoma *phylogeny and speciation

**Figure 2:**
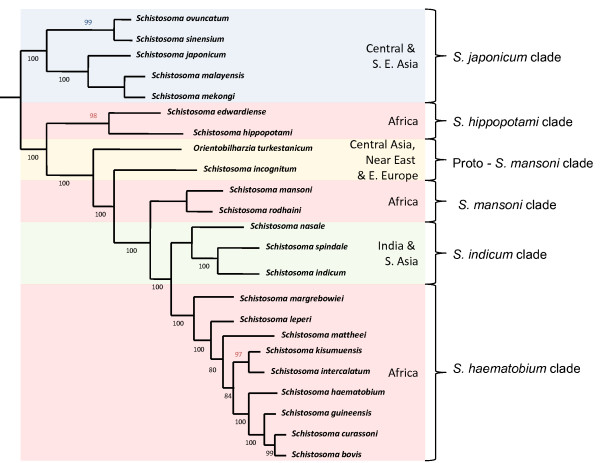
**Summary schematic phylogeny of the interrelationships of members of the species within the *Schistosoma* genus estimated with a Bayesian analysis of combined partial *lsrDNA*, complete *ssrDNA *and partial *cox1*.** Nodal support indicated as posterior probabilities and bootstrap percentages (n = 2000) from maximum parsimony analysis. This tree also indicates the four main clades and the two referred to in this study, which in the past have been classified on their egg morphology and both intermediate and definitive hosts: the *S. japonicum* group (*S. sinensium, S. ovuncatum *(inferred from partial* lsrDNA*)* S. japonicum, S. malayensis, S. mekongi*) being basal to the* Schistosoma *group, and the* S. mansoni  *group (*S. mansoni, S. rodhaini*) being the first major split in the African clades, with the *S. indicum* group (*S. nasale, S. spindale, S indicum*) and the *S. haematobium* group (*S. margrebowiei, S. leiperi, S. mattheei, S. intercalatum, S. kisumuensis, S. haematobium, S. guineensis, S. curassoni and S. bovis*). The tree also illustrates the basal nature of Asian schistosomes, being ancestral to the African stock due to the relative positions of *S. hippopotami*, *S. edwardiense* (Inferred from partial *cox1*), *Orientobilharzia* and *S. incognitum*. (Adapted from [8-10])

Over the past 15 years, there has been an increase in the use of molecular tools to study the interrelationships between organisms, with the most robust phylogenies of the schistosomes and the schistosomatids being constructed in the past eleven years by several researchers and several new species being described [7-15]. Although a range of genetic markers have been used to construct these trees, the nuclear marker ribosomal RNA gene unit (18S, 5.8S, 28S, internal transcribed spacer region (ITS)) and a number of the mitochondrial genes have been used extensively in both phylogenetic and phylogeographic studies of the schistosomes [5-19]. Using a multiple gene approach has provided validity to the construction of robust phylogenies. More recently, research has seen a move towards the use of mitochondrial markers to construct phylogenies and a standard phylogeny of the genus *Schistosoma *has been established and referenced in the literature [8-15] (Figure 2).

Although the phylogeny of the schistosomatids shows the schistosomes to be monophyletic, the genus *Schistosoma *is suggested to be split into six defined clades which correlate with the different geographical distributions of the parasites [7,8,11]. The species that form the *Schistosoma japonicum *complex (*S. sinensium, S. ovuncatum, S. japonicum, S. malayensis *and *S. mekongi*) appear basal on the tree, with the position of this clade providing strong evidence for the Asiatic origins of this parasite, suggesting an oriental origin and its colonisation of both the eastern and south eastern provinces of Asia [1,4,7,9].

The second major split in the phylogeny encompasses the species that are parasites found throughout Africa. However, there appears to be several distinct clades within this major split, with the most basal lineage described here as the *S. hippopotami *clade, containing both *S. hippopotami *and *S. edwardiense*, which were both described from the vascular system of hippos from western Uganda [10]. The next division within the phylogeny, termed as the proto-*S. mansoni *clade, has been shown to be basal to the true African species and is represented by two species, *Orientobilharzia turkestanicum*, which is now known to belong to the genus *Schistosoma*, and *S. incognitum *[8]. Both species are found in southern and western Asia, throughout the Middle East, while *O. turkestanicum *has also been found in Eastern Europe utilising red deer as a definitive host in particular foci in Hungary [11]. This again adds weight to the argument of an Asian origin for the African schistosomes and may even give an insight into speciation events that occurred before the colonisation of the African continent [[4,7,8] and [15]].

The African schistosomes form two distinct clades, the *S. mansoni *clade (*S. mansoni *and *S. rodhaini*) and the *S. haematobium *clade (*S. margrebowiei, S. leiperi, S. mattheei, S. intercalatum, S. haematobium, S. guineensis, S. curassoni, S. bovis *and *S. kisumuensis*). However, sitting between the two major African clades is the *S. indicum *group (*S. indicum, S. spindale *and *S. nasale*) which are found throughout southern and western Asia suggesting a reinvasion of the Asiatic subcontinent [4,5,7,8,12,15].

### Evolution of the schistosome mitochondrial genome

Mitochondrial genes have been used to facilitate the molecular analysis of the relationship between different species, because they tend to have higher mutation rates than nuclear markers and exist in high numbers. This has consequently led to their extensive exploitation for studying phylogenetic relationships and genetic variation in these parasites [17,20,21]. As discussed previously, most of the widely accepted ideas and concepts of *Schistosoma *phylogeny have primarily been based on the alignments of multiple gene sequences from a handful of genes such as *cox1, cox2, nad4, rrnL and rrnS *[8,10,15,20-22]. However, recent work has focused on gene order arrangement around the circular genome of the mitochondria, to utilise these molecular changes as phylogenetic markers [19]. Mitochondrial gene rearrangements are considered to be rare evolutionary events and the mutual differences that can be detected or observed among groups of organisms are thought to be indicative of shared ancestry [22,23].

**Figure 3:**
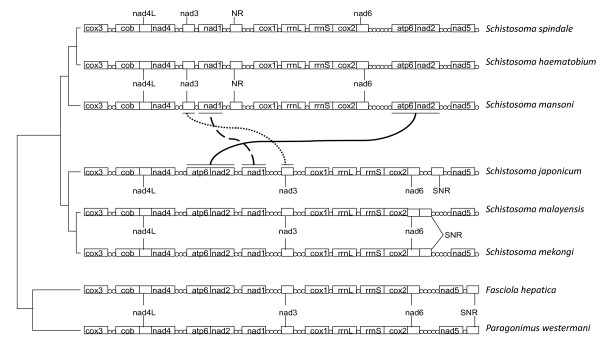
**Gene order rearrangements within the *Schistosoma* mitochondrial genome.** The Asian schistosomes from the S. japonicum complex appear to have retained the general digenean gene order in their mitochondrial genomes when compared to other flukes such as *Fasciola* and *Paragonimus*. However, major gene order changes in the African clades (*S. mansoni and S. haematobium*) and the *S. indicum* group are apparent. The phylogeny is a maximum likelihood tree estimated from complete 18S rDNA and gene order rearrangements in mitochondrial genomes provide characters for estimating positions on the phylogeny. Where the cytochrome oxidase genes are cox1, cox2, cox3; NADH dehydrogenase genes are nad1, nad2, nad3, nad4, nad4L, nad5, nad6; ATP synthase gene is atp6, cytochrome b gene is cob, rrnL is 16s, rrnS is 12s SNR, is the short noncoding region and the circles represent tRNAs (Adapted from [22-24]).

Currently, there are complete mitochondrial genomes available for six species of schistosome including *S. japonicum, S. mekongi, S. malayensis, S. spindale, S. mansoni *and *S. haematobium *[22-24]. Le *et al*. [23] illustrated that the mitochondrial genomes of *S. japonicum *and *S. mekongi *displayed the same gene order as each other and that of other digenea and cestodes [22]. However, *S. mansoni *shows several differences in gene order compared to the species from the *S. japonicum *clade. The *S. mansoni *type rearrangement was also seen in *S. haematobium *and *S. spindale *(Figure 3) [22]. This provided yet further evidence for the Asian ancestry of the schistosomes, and gave weight to the concept of the reinvasion of Asia by the *S. indicum *group from Africa [7,22,24]. This unique gene order rearrangement in the African schistosomes and their descendants is of particular interest when considering the phylogenetic position of *O. turkestanicum *and *S. incognitum *and their geographical ranges. These species may give insights into when and where these genomic changes occurred during the invasion of Africa and the radiation of the *Schistosoma *[22].

### The schistosome genome and karyotype evolution

All members of the *Schistosoma *genus are normally diploid and have eight pairs of chromosomes of which seven pairs are autosomal and, unlike most other trematodes, a pair of heterogametic sex chromosomes (male-ZZ and is female-ZW) [6,21,25,26], though a tentative triploid has been reported [27]. The current assembly of the *S. mansoni *and *S. japonicum *genome has shown that the nuclear genome is approximately 360 megabases, 40% repetitive, with the other 60% representing single copy sequences or small gene families, composed from approximately 11, 809 genes [28].

**Figure 4:**
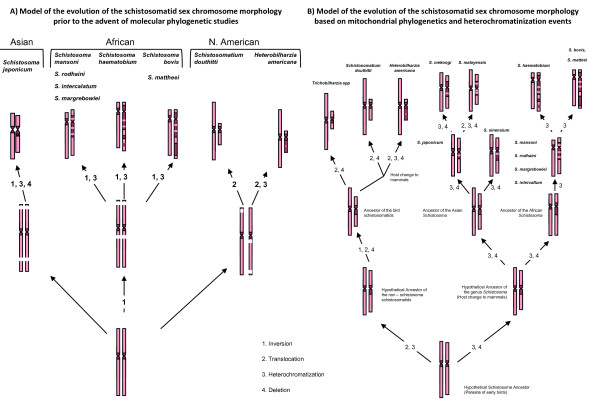
**Two models on the evolution of sex chromosomes of the schistosomatids**. A) Short and Grossman's (1981, 1983) Ideograms proposing the evolution of the sex chromosomes to have followed a similar trend to the rest of the genome, arising from a hermaphrodite ancestor. Two homologous chromosomes that contained the genes for sex determination began to diverge as a result of reduced recombination and gave rise to the ancestors of each schistosomatid group independently. B) Based on the mtDNA data and morphology of the chromosomes, a plausible model of sex chromosome evolution can be put forward suggesting that heterochromatinization events are the major factor in the differentiation of the sex chromosomes in the schistosomatids but also that the pattern of morphological differentiation shows the same relationship as the mitochondrial genome data.

Short [29] compared the C banded pattern of the chromosomes in the African schistosomes, and found that these species all displayed very similar patterns except for the patterns on the W chromosome. The heterochromatin composition on the W chromosome differed considerably between species. The centromeric area and proximal part of the long arm of the W chromosomes of *S. mansoni, S. rodhaini, S. intercalatum *and *S. margrebowiei *were shown to be heterochromatic. In *S. haematobium*, 3-5 differential staining heterochromatic bands were identified along the long arm of the W chromosome in addition to a paracentromeric block of heterochromatin (Figure 4) [30]. However, *S. bovis *and *S. mattheei *differed from the other species; *S. bovis *had a large euchromatic gap between two blocks of heterochromatin with a proportionally shorter, terminally located, euchromatic region. This was also true for *S. mattheei *except that the euchromatic gap was smaller than that in *S. bovis *[29]. When compared with the Asian schistosomes, there appeared to be many similarities with the African species in chromosome number and morphology (Figure 4).

Short and Grossman's studies [26,29,30] led them to theorise that there was a polyphyletic origin of the structure of the schistosome genome, and described two separate origins for the African and Asian species. However, Hirai *et al*. [21] compared the C banding patterns of the *S. japonicum *complex with *S. mansoni *and *S. haematobium*, and found lower heterochromatization throughout the karyotypes and lower recombination rates through chiasma frequency in the Asian species [31]. An intermediate between the Asian and African karyotype patterning was also identified in the karyotype of *S. sinensium*. This species showed the highest level of heterochromatization on the sex chromosomes resulting in the largest W chromosome compared to the Z of all the Asian species, but also has the same C banding pattern as the African *S. mansoni *on chromosome 2 [21]. These findings lead to the hypothesis that the African schistosomes were derived from an Asian *S. japonicum *like ancestor as also suggested by Snyder and Loker [13] and were not polyphyletic as suggested by Short [29] (Figure 4).

The widely accepted and universal theory suggests that sex chromosome evolution occurs by a gradual arrest of recombination between two homologous chromosomes, followed by the genetic decay of one of these chromosomes that will gradually become the gametotype of the heterogametic sex [32,33]. This process may be triggered by the favourable disequilibrium between sex-determining and a sexually antagonistic locus which acts as a selective pressure to reduce recombination of these loci [32,33] (however see Ironside [34] for an alternative view). As a result, there is an eventual loss of functional genes from the heterogametic sex chromosome due to the gradual mutations or rearrangements [32,33,35]. This has been suggested to be driven by several processes including Muller's ratchet, genetic hitch hiking and even background selection, all of which relate to population size, density and distribution. In spite of a lack of molecular knowledge, Short (1983) [29] touched on these concepts and suggested that during the early evolution of sex chromosomes in schistosomes, the homologues were originally morphologically identical; with subtle differences in only a few genes and as sex determining genes accumulated on a specific homologous pair of chromosomes, the potential for sex chromosomes arose. Differentiation of the true Z and W chromosomes in these worms depended on some mechanism to prevent crossing over of regions with sex determining genes in order to reduce free recombination [29]. Grossman *et al*. [30] suggested that it may have been possible that the heterochromatin activity prevented crossing over between the differential segments of the ancestral Z and W and suppressed the expression of particular genes, allowing the development of true sex chromosomes in the schistosomes, which was also indicated in investigations by Hirai *et al*. [31] on chiasma frequencies in parasitic flukes.

Although the autosomal karyotype of the African schistosome species is indistinguishable the sex chromosomes are markedly different in both size and structure displaying species specific characteristics in heterochromatic patterns on the W chromosome [29]. This is also true of the *S. japonicum *group being distinguished by metacentric sex chromosomes with little heterochromatin. The Asian schistosomes show the W chromosome to be much smaller than the Z chromosome, conforming to the hypothesis that the W chromosome is degenerate resulting from the lack of recombination [31,35]. However, the W chromosome is relatively much larger than the Z chromosome in the African species, although all chromosomes are considerably smaller than those of the Asian schistosomes [25]. This alteration in size is the consequence of the increased heterochromatization of the W chromosomes in African species and may be used to indicate regions where there is suppression of recombination [31,35,36]. These heterochromatic bodies have occurred in the respective areas of recombination in the *S. japonicum *group and *S. mansoni*, suggesting that there may be some selection for heterochromatization of the W chromosome as an adaptation to suppress recombination with the homologous regions of the Z chromosome [25-30] (Figure 4).

However, when the cytogenetic data from both Short [29] and Hirai *et al*. [25] are combined with the "true" phylogeny adapted from Webster *et al*. [8] an alternative theory of the evolution of sex chromosomes can be proposed, which conforms to the out of Asia hypothesis that Hirai *et al*. [25] eluded to (Figure 4). The Asian schistosomes appear to have lower levels of heterochromatization on the sex chromosomes than do the African schistosomes. In the Asian clade, *S. japonicum *has a relatively simpler set of sex chromosomes with a small W chromosome and a larger Z chromosome. Both *S. mekongi *and *S. malayensis *show a similar pattern of heterochromatization as *S. japonicum *but have a larger mass of heterochromatin on the W, suggesting these species were derived from this type of karyotype. As noted by Hirai *et al*. [25], *S. sinensium *appears to have characteristics of both Asian and African chromosomes, and although the W chromosome is smaller than the Z chromosome, the W chromosome does show a very similar pattern of heterochromatin on the p and q arms, as is found in *S. mansoni*, leading to the suggestion that *S. sinensium *has retained the ancestral characteristics of both African and Asian species. Therefore, it is plausible that *S. japonicum *diverged from a basal *S. sinensium*-like stock and the African like characteristics were lost as the *S. japonicum *group arose. The African clade shows several marked differences, with the Z chromosome being smaller than the W chromosome, and W chromosome having large heterochromatic masses; however both the sex chromosomes are markedly smaller than those observed in Asian species. The *S. mansoni *type sex chromosomes appear to occur in both the *S. mansoni *and the *S. haematobium *clade with all other forms appearing to be derived from it, suggesting this to be the ancestral form that would have originally invaded Africa. Both *S. bovis *and *S. haematobium *show higher levels of heterochromatin in the W chromosome than that of *S. mansoni*. This could have happened as a gradual heterochromatization event during speciation to give rise to the two different forms or may have resulted from an inversion event on the W chromosome of the *S. mansoni*-like ancestor, involving inversion of the large heterochromatin mass into the pseudoautosomal region. Additionally this could have happened as the *S. haematobium *type karyotype arose from such an inversion followed by a heterochromatization event that produced the *S. bovis *type sex chromosome karyotype (Figure 4).

### Genomic insights into the evolution of *Schistosoma*

**Figure 5:**
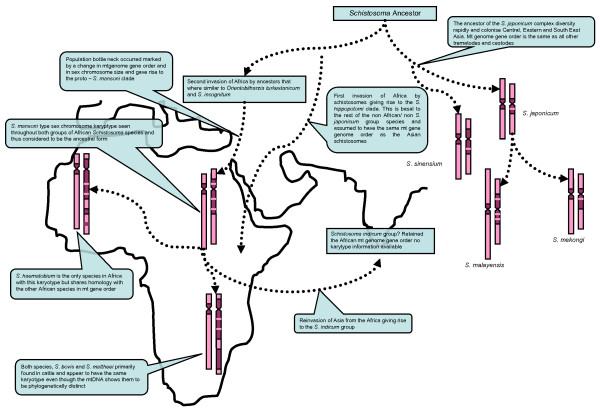
**Semi-schematic summarizing a phylogeography and genome evolution of *Schistosoma *with respect to the evolutionary biogeography of the parasites showing that the genus arose in Central Asia in rodents during the Miocene**. The *S. japonicum *lineage radiates in rodents in China and Southeast Asia. *Orientobilharzia *acquires artiodactyls as hosts in Central Asia sharing homology with an ancestor that enters Africa during the late Miocene and gives rise to the *S. haematobium *and *S. mansoni *lineages. The Pliocene large mammal radiation in Africa triggers the divergence of several lineages of *Schistosoma *utilizing Artiodactyla. The Plio-Pleistocene large mammal divergence into Asia and the emergence of Bovidae then drives the divergence of the *Schistosoma indicum *lineage from one of the African artiodactyle *Schistosoma *clades. *Schistosoma *of bovids establish on the Indian subcontinent and in Southeast Asia but apparently less so in Central Asia. In Southeast Asia, both *S. incognitum *and *S. spindale *undergo near isochronous colonization and radiation from India. The schematic indicates the evolutionary signals that have been produced by genomic data to provide the evidence of the Asian origin of the schistosomes. The ancestor of the African schistosomes shared homology with *Orientobilharzia *and *S. incognitum *and a genetic bottleneck occurred, as indicated by the reduction in size of the sex chromosomes and fixed the mt genome rearrangement as the schistosomes moved into Africa. The African schistosomes evolved from a *S. mansoni*-like ancestor with all African parasites showing the same gene order in the mt genome and all showing karyotypes to be derived from the *S. mansoni *type, a trend also seen in the *S. indicum *group as it reinvaded Asia (Adapted from [1]).

By combining the mitochondrial data with that of the cytogenetic data, and Attwood's *et al*. [1] description of the movement of schistosomes, a plausible theory can be constructed to aid in the understanding of the evolution of these parasites (Figure 4 &5):

1) *Asian origin*: mitochondrial, nuclear and cytogenetic data suggest an Asian origin with the *S. japonicum *group appearing to be basal to other members of the genus [8,15,16,20]. These observations suggest that *S. sinensium *is basal to the *S. japonicum *group and the cytogenetic data show it to have characters of both African and Asian species. Although the shape and structure of the sex chromosomes within *S. sinensium *corresponds to that of the Asian *S. japonicum *clade, in that the Z chromosome is markedly larger than the W chromosome, they do have high levels of differentiation similar to that in the African species. Chromosome 2 also shows marked similarities to that of the African species and in fact this worm has similar egg morphology to the *S. mansoni *group. With this in mind, it is possible to suggest that a *S. sinensium-*like genome is most likely to be the ancestral state, with *S. japonicum *diverging forming a sister group. Also, *S. ovuncatum*, the sister taxon of *S. sinensium*, has an egg morphology which closely resembles that of *S. mekongi *rather than the *S. mansoni *type of morphology seen in *S. sinensium *[9]. This again suggests that other African type characteristics could have been soon lost in the radiation of the Asian lineages. The similarities seen between *S. mekongi *and *S. malayensis *may also suggest recent divergence away from the ancestral state, with the sex chromosomes appearing more divergent from those found in *S. japonicum *but the whole genome sharing the majority of traits. (Figure 4).

2) *The invasion of Africa*: According to the molecular mitochondrial data the invasion of Africa happened on at least two separate occasions. The first event occurred when the ancestors of the hippos moved across the land bridge between Africa and Asia utilising the large freshwater networks that eventually would have given rise to the Nile basin and the Mediterranean Sea [10]. This would have evolved into the *S. hippopotami *clade which will have probably become isolated and diversified in Africa after the last ice age [1,10] The *S. hippopotami *clade shares a common Asian ancestor, with *O. turkestanicum* and *S. incognitum *subsequently basal to the African stock of *Schistosoma *that gave rise to the *S. mansoni *and *S. haematobium *clades [8,13]. However, due to the lack of a complete mitochondrial genome or a full karyotype for these species, it is only possible to speculate what their ancestral genomic state may have been. Comparison of the mitochondrial genome of the Asian and African species shows that there are substantial gene order rearrangements. By studying this in *O. turkestanicum *and *S. incognitum*, it may be possible to gain some insight into where and when these changes happened and under which circumstances such mutations arose. Interestingly, this also coincides with the marked difference in the sex chromosomes between the Asian and African clades. The African species appear to have smaller chromosomes than the Asian stock but their sex chromosomes are much more differentiated from each other and, due to higher levels of heterochromatization, the W chromosome has become much larger than the Z chromosome. Both genomic changes in the mitochondria and reduction in sex chromosome size can often be associated with population bottlenecks which would coincide with the movement of parasites from Asia and the colonisation of Africa during the mid-Miocene with radiation of mammals from Asia into Africa [1]. It is also important to note that when the karyotypes of the African species are compared with the phylogenies constructed by Lockyer *et al*. [15] and Webster *et al*. [8] the majority of species have the *S. mansoni *type karyotype and sex chromosome patterning, suggesting that this was the ancestral state of the genome of the African stock (Figure 4). However, there is much more variation of the sex chromosome patterning in the *S. haematobium *clade although the *S. mansoni *type still appears to be the most common and is found in *S. margrebowiei *which is basal to this clade, and in *S. intercalatum/S. guineensis *which are situated within the clade. This suggests that the variation seen in the patterns of sex chromosomes are variations on the *S. mansoni *karyotype and may have occurred relatively recently. This corroborates the molecular data that suggest a radiation in the *S. haematobium *group within the past 2-5 million years, but with both *S. mansoni *and *S. haematobium *diverging some 15 million years previously [1](Figure 4).

3) *Reinvasion of Asia*: based primarily on the molecular data provided by Lockyer *et al*. [15] and Webster *et al*. [8] and the complete mitochondrial genome of *S. spindale *[18], there are substantial affinities between the *S. indicum *group and the African stock that suggests an African ancestry. Unfortunately, like *O. turkestanicum *and *S. incognitum *there is currently no data on the karyotypes of the *S. indicum *group but two scenarios could be suggested based on the evidence from the African stock. 1) It is most likely that these species show the *S. mansoni *type of sex chromosomes or 2) because of the recent divergence may show drastic heterochromatic changes in sex chromosome patterns, as have occurred in *S. bovis, S. mattheei *and *S. haematobium*. However, this is purely speculation and needs further investigation (Figure 4). Interestingly, the reinvasion of Asia was not accompanied by any definitive host switching into primates and carnivores with *S. indicum, S. spindale *and *S. nasale *continuing to parasitise ungulates and rodents [1,8].

By considering the genomic evidence a general scenario for the history of the schistosomes can be put forward. The genus *Schistosoma *arose in Asia from an avian schistosomatid, probably as parasites of murid rodents, possibly 60-70 million years ago, eventually followed by another host shift to utilise ungulates approximately 20 million years ago during the mid to late Miocene [1] (Figure 5). This was probably the trigger for the diversification of the *S. japonicum *group, as this also coincided with the diversification of their pomatiopsid hosts [13]. The *S. japonicum *group share distinct genomic similarities with the avian parasites, including mitochondrial gene order and the relative size ratio between the Z and W chromosome. This indicates that the *S. japonicum *group retained some of the "ancestral" type genome traits. It is also important to note that *S. japonicum *has over 40 different hosts which may be a strategy that would have allowed the proto forms of the *S. japonicum *group to retain ancestral genome characteristics [4,6]. If this is the case, it could be suggested that the variation seen in molecular data and from cytogenetics may have been influenced by the specialisation to specific hosts (Figure 5).

During this host switch from rodents to ungulates, it has been suggested that *O turkestanicum *represents an offshoot of the Asian rodent parasites that began to specialise in utilising artiodactylians in central and eastern Asia [1,37]. This coincides with the divergence of ungulates across Europe, Asia and Africa that took place during the Miocene. If this is the case, then the mass movement of ungulates between Africa and Asia as the Tethyan seaway closed would have given a viable route for the schistosomes to invade Africa [1,13] (Figure 5). This also corresponds with RFLP data, suggesting that *S. mansoni *was in Africa before the end of the Miocene [9,38]. However, during the invasion of the Middle East and Africa, population bottlenecks would have occurred as a result of the lack of a pomatiopsid intermediate host, forcing a host change to planorbid snails. The reduction in the sex chromosome size indicates that such a bottleneck may have occurred and would have been the event that also fixed the mitochondrial gene order change in the ancestral stock of the African schistosomes [39] (Figure 5).

Both the *S. mansoni *type karyotype and mitochondrial genome represent the proto African schistosome with all other species appearing to be derived from it. With the radiation of both the *S. mansoni *and *S. haematobium *groups, this allowed species to utilise not only ungulates and rodents but also primates as definitive hosts and eventually allowed them to specialise in snails of the *Biomphalaria *(*S. mansoni *group) and *Bulinus *genus (*S. haematobium *group). Attwood *et al*. [1] suggested that reinvasion of Asia by the ancestral stock of the *S. indicum *group probably occurred about 2-3 million years ago as large ungulates migrated across the Sinai-Levant dispersal tract between the Pliocene and Pleistocene, and the emergence of the Bovidae during this period, probably facilitated diversification of the *S. indicum *group giving rise to both *S. nasale *and *S. spindale *(Figure 5).

## Conclusion

With the absence of fossil evidence for the schistosomes, it would be impossible to infer theories on their evolution without the use of genomic data from both molecular and cytogenetic techniques. Although some gaps still remain in the data, it is possible to speculate on the causes of those changes at the genomic level and to provide strong evidence for the Asian origin of these parasites (Figure 5). With the completion of the *S. mansoni *and *S. japonicum *genome and the first version of the assembly available in the public domain, it is now possible to swiftly develop genetic markers that can be utilised to understand the evolutionary history of the these parasites [40]. Such markers, including gene sequences, microsatellites and SNPs, must be used together if an accurate account of schistosome evolution is to be inferred [6,8,41]. In the broader context, the data generated using molecular and genetic techniques not only gives an insight into the evolutionary history of these organisms but also into the population biology of these parasites which consequently has a direct impact on species identification, biology, and disease epidemiology.

## Abbreviations

*cox1*: Cytochrome oxidase 1; *cox2: *Cytochrome oxidase 2; MYA: Million years ago; *nad4: *NADH dehydrogenase; *lsrDNA: *Large subunit of ribosomal DNA; *ssrDNA*: Small subunit of ribosomal DNA; RFLP: Restriction fragment length polymorphism; RNA: Ribose nucleic acid; rDNA: ribosomal DNA; *rrnL: *mitochondrial large subunit of ribosomal DNA; *rrnS: *mitochondrial small subunit of ribosomal DNA; SNPs: Single nucleotide polymorphism.

## Competing interests

The authors declare that they have no competing interests.

## Authors' contributions

SPL wrote the manuscript and was responsible for conceiving the new proposed theories and hypotheses based on current genomic knowledge of the schistosomes. HH developed the theoretical aspects of chromosome structural evolution and helped with the draft of manuscript. JEI provided expertise into general sex chromosome evolutionary theory and critically reviewed manuscript before publication. DAJ provided expertise and opinion on schistosome genomics and molecular evolution and helped with the draft of the manuscript. DR provided expertise on schistosome biology and population genetics and critically approved several drafts of the manuscript before publication. All authors read and approved the final version of the manuscript.
